# Estimating Competition between Wildlife and Humans–A Case of Cormorants and Coastal Fisheries in the Baltic Sea

**DOI:** 10.1371/journal.pone.0083763

**Published:** 2013-12-30

**Authors:** Örjan Östman, Maria K. Boström, Ulf Bergström, Jan Andersson, Sven-Gunnar Lunneryd

**Affiliations:** Department of Aquatic Resources, Swedish University of Agricultural Sciences, Öregrund, Sweden; University of Lleida, Spain

## Abstract

Cormorants and other wildlife populations have come in real or perceived conflicts with humans over exploited fish stocks. From gut contents of cormorants, and using an extension of the Catch equation, we estimated the degree of short term competition between great cormorants and coastal fisheries in two areas along the Swedish Baltic Sea. Cormorants consumed 10 and 44%, in respective area, of the fish biomass of six fish species harvested by humans; eel, flounder, herring, perch, pike, and whitefish. On average, cormorants consumed smaller individuals than harvested in fisheries. But for perch, cod and flounder, cormorants consumed harvestable sized fish corresponding >20% of human catches. Our competition model estimated the direct decrease in fisheries catches due to cormorant predation to be <10% for all species except flounder (>30%) and perch (2–20%). When also including the indirect effects of cormorant predation on smaller fish that never reached harvestable size, the estimated decrease in fisheries catches at least doubled for perch (13–34%) and pike (8–19%). Despite large uncertainties, our model indicates that cormorants may locally have a direct impact on human catches of at least flounder, and when incorporating indirect effects also on perch and pike. The study indicates that the degree of competition between cormorants and humans varies substantially between areas. We also included economical values in the model and concluded that for the commercially most important species, eel and cod, the estimated economic impact of cormorants on fisheries was low.

## Introduction

Wildlife populations can compete with humans over shared resources. In many aquatic ecosystems worldwide there is a potential competition between fish-eating birds like cormorants and humans for stocks exploited by fisheries [1], [2], [3]. Along the Swedish Baltic Sea coast great cormorants (*Phalacrocorax carbo sinesis*) has expanded from a few hundred birds in the 1950’s to 42 000 breeding pairs in 2009 [4]. They consume 400–600 g of fish per individual and day [5], [6], so fish consumption by cormorants has multi-folded along the coasts of the Baltic Sea. At the same time catches, and sometimes catches per unit effort, of species exploited by fisheries have declined [2], [7]. This has raised an issue of possible competition over fish resources between cormorants and coastal fisheries in the Baltic Sea. Commercial fisheries along the Swedish coast of the Baltic Sea Proper primarily target cod (*Gadus morhua*), eel (*Anguilla anguilla)* and salmon (*Salmo salar*). Herring (*Clupea harengus*), flounder (*Platichthys flesus*), perch (*Perca fluviatilis*), pike (*Esox lucius*), pikeperch (*Sander lucioperca*), and whitefish (*Coregonus lavaretus*) are complementary resources [8]. For the recreational fishery, perch, pike, and herring are the main targeted species, but locally also whitefish and sea-trout (*Salmo trutta trutta*) [9].

There are studies showing long-term negative associations between cormorant abundance and harvest rates in fisheries, especially between double-crested cormorant and yellow perch in North American lakes [1]. Other studies show non-significant effects of cormorants on fisheries in the Baltic Sea region and elsewhere [10], [11], [12], [13]. In the Baltic Sea there are indications that cormorants locally may have a negative impact on the long term abundance of perch [2], [14]. For other species the influence of cormorants on long term dynamics is uncertain or insignificant [14], [15].

The population dynamics of coastal fishes depend on several other factors than cormorant predation such as the abiotic environment, predation from other piscivores, eutrophication and habitat changes [14], [16]. Although cormorants may have small impact on population dynamics they can consume large quantities of fish also exploited by fisheries [17], [18], [19]. Even though cormorants consume in parity or more than humans there are two factors that may lower competition. First, cormorant and fishing mortalities may be low relative to other mortalities [20]. Second, cormorants tend to feed mainly on fishes smaller than humans target [17], [19], [20], [21]. On the other hand, the cormorants’ predation on small fishes may impact fisheries yields if fewer fishes reach sizes large enough to be harvested [22]. Whereas several studies have made associations between cormorant abundances, fish abundance and landings, few or none has tried to estimate the potential impact of cormorants on human catches.

Here we have studied gut content from culled great cormorants in two areas along the Swedish Baltic Sea coast. Both areas inhibit 1500–2000 breeding pairs over a coastal stretch of 50 km, but the two areas represent two ends of the fish community spectrum in the Baltic Sea Proper. The Karlskrona archipelago has a relative high abundance of piscivorous fish like perch, pike, and cod, and fishery aims towards cod and pike. In Mönsterås archipelago the fish community is dominated by benthivorous and planktivorous fishes, e.g. cyprinids and sticklebacks [7], and fishery mainly aim toward eel. By using an extension of the Catch equation (e.g. [23]) we quantitatively estimated the competition between cormorants and fisheries for a broad range of species. An advantage of this approach is that short-term competition can be estimated from only consumption levels by cormorants and fisheries and mortality rates. Our aims were threefold: 1) Identify for which fish stocks there is a potential direct competition between fisheries and cormorants over harvestable sized fish. 2) Study the importance of an indirect competition from cormorants feeding on small fish. And 3) investigate whether the potential competition between cormorants and fisheries varies between areas as a consequence of differences in fish communities.

## Materials and Methods

### Ethics Statement

The great cormorant is protected according to the EU Bird directive (79/409). The cormorants in this study were hunted under allowance of culling as a protection measure of fishing gears in Mönsterås archipelago, provided by the County administrative board of Kalmar (permit 218–1897-10), and in Karlskrona archipelago by the County administrative board of Blekinge (permit 218-6352-09). The hunting was done by two local fishermen. All cormorants were shot with shotgun according to Swedish hunting legislation and not in any conservation area. No wounded cormorant was reported. During hunt under breeding season (May–June) only juvenile birds and none-breeding birds were collected with permission of a scientific hunting quota (of maximum 600 individuals) issued by the Swedish Environmental Protection Agency, permit 412-1680-09. The whole study was judged and approved by the Ethical Board on Animal Experiments of the County Court of Linköping, Sweden, permit E1809-09.

### Length Distributions of Fish in Cormorant Diets

We studied gut content of 524 great cormorants in two areas along the Swedish Baltic Sea coast. In the Karlskrona archipelago, in southeast Sweden, we collected cormorants during one and a half year, and in the Mönsterås archipelago, on the Swedish east coast, we collected cormorants during one year (Table S1, Fig. S1). Both archipelagos have brackish water and is characterised by many small islands with rather shallow waters in between, and thus, dominated by demersal fish. These archipelago are important spawning (herring) or foraging grounds for immature (cod, flounder) open sea fish species.

Cormorants were shot outside colonies, i.e. foraging, resting or flying birds, in the archipelagos of Karlskrona (ICES rectangle 4160) and Mönsterås (northern part of rectangle 4261 and southern part of rectangle 4361, Figure S1). Each rectangle covers approximately a 50 km stretch of the coast. This corresponds well to the maximum foraging distance of breeding birds [24], [25]. Hence, most of the fish were consumed within the studied coastal areas. Total numbers of cormorants culled per month and archipelago are available in Table S1. A detailed description of handling, preparation and estimation of cormorant gut content and size distributions is available in [26]. Based on counts and length measurements of otoliths recovered in the cormorant stomachs we estimated the biomass of different fish species per centimetre interval. Unidentified otoliths and otoliths too eroded to estimate lengths were excluded before estimating diet composition. Estimates were made for May-September and October-April separately motivated by a seasonal change in the shallow-water fish community [27]. There is also a separation of breeding and non-breeding cormorants between the two periods. The relative contribution in biomass of different fish species per centimetre interval was calculated as the estimated biomass of a species and size class in relation to the total estimated biomass of fishes in all sampled cormorants for each period and site.

To estimate the total biomass of fish consumed by cormorants we calculated the total expected fish consumption from bioenergetic models (Table S2). For each adult during egg incubation we used the daily fish consumption of 238 g/day for 30 days estimated from a cormorant colony in the southern Baltic Sea [5]. They also estimated daily intake to 316 g/day when rearing small chicks (10 days), and 588 g/day when rearing downy chicks (40 days). These estimates also include the fish fed to the chicks, assuming one chick per adult. For both adults and juvenile in the post-breeding phase (100 days, ending 30 September) we used the estimated intake of 540 g/day from [6]. Also 540 g/day and individual was used for cormorants October-April, irrespective of age. Local fishermen and ornithologists estimated the number of breeding cormorants to 1750 pairs in Karlskrona archipelago [4], and 1500 pairs in Mönsterås archipelago [28]. There were no counts of non-breeding individuals in the areas, but other studies indicate around one non-breeder per breeding pair [29]. There were no counts of cormorants in October-April from the areas. Cormorants need open waters for foraging and hence abundances should differ between years and areas depending on ice cover. In Denmark the total number of cormorants during October-April is on average around 35% of the total number of cormorants during the whole year [30]. The ice cover period is longer in the studied areas here than around Denmark, and hence, cormorants should be relatively less abundant here during October-April. We therefore scaled the relative abundance during October-April with average number of days with ice cover, assuming the relative abundance of cormorants during October-April is inversely proportionate to the ice cover period. The ice cover period along the Danish Baltic Sea coast is around 10 days per year [31]. Corresponding figures at Karlskrona and Mönsterås are 25 and 50 days per year, respectively [31]. This gave an estimated relative abundance of cormorants during October-April of 20% and 10% in Karlskrona and Mönsterås archipelago, respectively (Table S2).

Fish species found in cormorant guts also important for commercial or recreational fisheries in the areas were perch, pike, eel, flounder, herring and whitefish. We calculated the amount of fish consumed by cormorants in size classes large enough to be included in fisheries (harvestable size) using the minimum size limit by regulation in the Baltic Proper, or minimum size to be considered as marketable product [32], [33]. These are ≥20 cm for perch, ≥30 cm for whitefish, ≥18 cm for herring, ≥21 cm for flounder, ≥38 cm for cod, ≥40 cm for pike and ≥65 cm for eel.

### Overlap and Potential Increase of Fisheries Catches

Commercial catch data was collected from the logbooks from coastal fisheries, using nets, hooks, static gears and trawl nets [34] for the ICES rectangle 4160 for Karlskrona and 4361 for Mönsterås (we considered 4261 non-representative for this area due to high catches of cod and herring and low catches of eel and therefore omitted). For each species we calculated an annual average biomass harvested in commercial coastal fisheries between 2008–2010. Catches in recreational fisheries were estimated from national questionnaires performed in 2006 and 2009 [9]. These questionnaires do not present information at the spatial resolution used in this study and we downscaled the catches from larger coastal zones (ICES subdivisions) to the specific study areas. We down-scaled recreational catches by assuming the proportion of recreational catches in each ICES rectangle to the whole coastal zone was identical to the corresponding proportion of commercial catches. Recreational fishing for eel was closed in 2007 and set to zero.

To calculate the potential decrease in fisheries catches due to cormorant predation we used the catch equation [23]. The benefit of using the catch equation is that absolute densities do not have to be estimated. Instead it is based on consumption levels and mortality rates. The yield in fisheries (*C_f_*) and consumption by cormorants (*C_c_*) between time *t* and time *t*+1 is a function of number of fishes (*N_t_*) at time *t*, fishing mortality (*F*), cormorant mortality (*M_c_*) and other natural mortalities (*M_n_*). The catch equation then becomes:
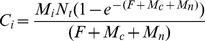
(1)where *C_i_* is fish consumed by either fisheries or cormorants and *M_i_* is the corresponding instantaneous fish mortality (i.e. F or *M_c_*). As *N_t_* must be identical for both *C_f_* and *C_c_* we get:



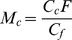
(2)We have estimates of *C_c_* and know *C_f_*. *F* is unknown but we can attain estimates from the literature or independent data [33], [35]. We insert eq. 2 into eq. 1 to estimate *N_t_*. To calculate the estimated fisheries yield (*C_F_*) in absence of cormorant predation on a fish species, our estimate of *N_t_* is inserted into eq. 1 so that:
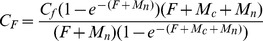
(3)


*M_n_* is unknown but estimates are available from literature or independent data [33], [35]. The R-script used for calculating (*C_F_*) is available in File S1. Note that the degree of competition is estimated from yields and mortality rates only and not stock size. Stock size only affects our estimate indirectly through the calculations of *F* and *M_c_*. To get number of fishes in human catches we divided total harvested biomass with the average body weight of fishes in commercial fisheries estimated from commercial catches [34]. Parameter estimates are presented in Table S3.

The calculations are associated with many uncertainties and to address this we calculated 95% confidence interval from the posterior distributions from 100 000 Monte Carlo simulations. We assumed all variables have a standard variation equal to one third of the parameter estimate (coefficient of variation, CV = 0.33), which gave reasonable confidence intervals for our parameters. The real dispersion for many of these parameters are unknown and by setting standard deviation equal to a fraction of the parameter value we keep variance neutral among variables. For example, 95% of the simulated values for a mortality of 0.2 will be in the range 0.07–0.33, and 95% of simulated values for a consumption of 200 000 kg, will be in the range 70 000–330 000 kg. Our aim is not to calculate dispersion estimates of our results as the fraction used is arbitrarily set. Instead the upper 95% percentile should be interpreted, given our assumptions, as the 2.5% likelihood of an impact of cormorants on fisheries catches higher than this value, and vice versa for the lower 95% percentile (2.5% probability of a lower impact). For the fish species not detected in the diet in Mönsterås archipelago we set standard deviation to a third of 2000. This was the lowest number of individuals needed to be consumed by cormorants to be detected in the diet.

### Indirect Effects of Cormorant Predation

Perch and pike are rarely moving >10 km during their life-span [36] and remain most of their life within the same coastal area. Whitefish is more motile, with migrations of 20–300 km, but is confined to the coastal zone and tributaries [36], [37]. Although eel displays an extreme migration, the yellow eel life stage is here considered as non-migratory. Hence for these species, the cormorant consumption on individuals smaller than harvestable sizes may indirectly affect local fisheries in later years.

To estimate the reduction in biomass of fish recruited into harvestable sized fish in later years, accounting for the cormorants’ consumption of small fish, we calculated a ‘Harvest equivalent’ measure:
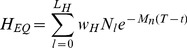
(4)
*w_H_* is the average individual weight at minimum harvestable size and *L_H_* is the minimum harvestable size. *N_l_* is the estimated number of fish consumed by cormorants in length class *l*, *M_n_* is natural mortality (other than cormorants), and *T-t* is the time (in years) for a fish to grow between length *l* and *L_H_*. Hence, *T* is age at length *L_H_*. In the case of eel, *H_EQ_* corresponds to the estimated impact of cormorants on total silver eel escapement as these eels migrate out of the system. The potential increase in fisheries yields of eels is then *H_EQ_*(1−e^−*F*^). For the other species (perch, pike and whitefish) we estimated the potential yields in weight (*H_PY_*) that could come from an increase in abundance of harvestable fish of *H_EQ_* as:
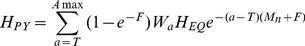
(5)F is fishing mortality, Wa is the weight at age a divided by weight at age T, and Amax is maximum age of a fish species. Here Amax = 30 which means the contribution at that age class to total harvestable biomass of a species was virtually zero. Species specific parameter estimates of wH, T, and Wa+A were obtained from fisheries independent surveys [33], available in Table S3. For perch, parameters could be estimated from surveys at Mönsterås and Torhamn in the Eastern part of Karlskrona archipelago, whereas for pike and whitefish samples were from the ICES subdivisions 25, 27 and 29 (Table S4). In eqn. 4 and 5, Mn and Wa are assumed to be constant and not related to densities. However, natural mortality is likely to increase and age specific body mass to decrease with population densities. To get an estimate of potential compensatory effects during increased densities we therefore calculated HEQ and HPY with doubling Mn and halving Wa as a response to increased fish densities from reduced cormorant predation.

### Economic Estimates

To estimate the monetary value of the cormorants’ catches we used the list price paid by wholesalers to commercial fishermen. Based on an exchange rate of €1 = 9 SEK, the average price per kilo fish over a year was: perch and whitefish €3.5, pike €1.8, cod €1.5, flounder €0.9, herring €0.5, and eel €10.4 [34]. The same values per kilo fish was used for estimating the marginal loss of economic value in commercial fisheries from cormorant consumption (price × [*C_F_* – *C_f_*]). However, wholesalers’ list price is a poor estimate of the economic value in recreational fisheries of perch, pike, cod, flounder and whitefish. For these species, we instead used the estimated average consumer surplus of recreational fisheries (total willingness to pay for fishing minus actual costs) divided by estimated total catches in recreational fisheries, which has been estimated to €4.4/kg [9]. This is a crude estimate across all recreational fisheries and is an average value, not a marginal value of catching another fish. But it at least provides some information about the loss of economic value to recreational fisheries from cormorants.

## Results

### Direct Competition

**Figure 1:**
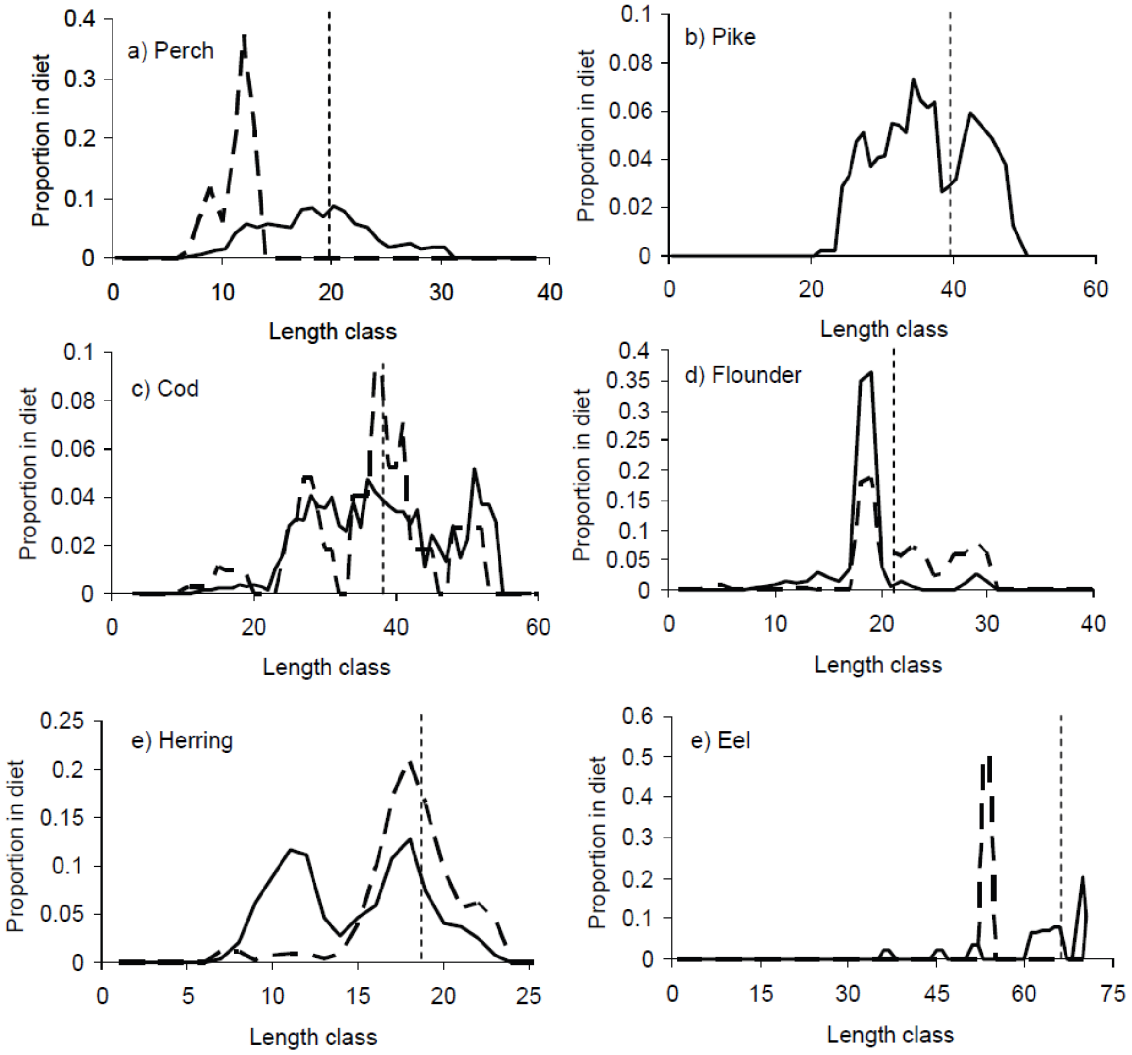
Proportion of biomass of different fish species per length class (cm) found in the gut of cormorants in Karlskrona archipelago (solid line) and Mönsterås archipelago (dashed line). Hatched vertical lines indicate the size at which respective fish species is recruited to human fisheries. Lines represent moving averages over three centimetres.

The cormorants’ estimated total consumption of cod, flounder, herring, perch, pike, and whitefish together was 44 and 10% of catches in commercial and recreational fisheries in Karlskrona and Mönsterås archipelagos, respectively (Table 1). Cormorants on average fed on fish smaller then harvestable size in fisheries, i.e. the major proportion of fish consumed is to the left of the minimum size in human fisheries in Fig. 1. The cormorants’ consumption of harvestable sized fish was 14 and 5% of the total human harvest in respective archipelago but differed between species (Table 1).

**Table 1:** Total estimated consumption of fishes harvested by humans and cormorants both in total (including all size classes) and for harvestable sized fishes (i.e. sizes large enough to be included in human fisheries) at Karlskrona (K) and Mönsterås (M) archipelagos.

Species	Total cormorant consumption kg/year		Cormorant consumption of large fishes kg/year		Commercial catches kg/year		Recreational catches kg/year		% Relative human catches		%Harvestable sized fish relative human catches		Commercial value of fish consumed by cormorants	
	K	M	K	M	K	M	K	M	K	M	K	M	K	M
Perch	99419	167	48663	0	1960	680	80100	5060	121%	3%	59%	0%	172	0
Pike	93274	0	25727	0	2627	1545	260800	20720	35%	0%	10%	0%	46	0
Cod	117996	22856	52103	4783	115745	16894	24400	5200	84%	103%	37%	22%	76	7
Flounder	65551	50839	5192	27323	1031	4748	4500	13680	1185%	276%	94%	148%	5	25
Herring	23579	34929	5641	18866	419976	971343	2520	11820	6%	4%	1%	2%	3	10
Eel	12984	2638	0*	0*	8905	49003	0	0	146%	5%	0*	0*	0*	0*
Whitefish	2152	0	556	0	2529	918	28050	4235	7%	0%	2%	0%	2	0
Sum	414955	111430	139541	50972	552773	1045131	400370	60715	44%	10%	14%	5%	304	4

Human catches are divided into reported commercial catches and estimated catches from recreational fishing. The next two columns show the cormorants’ total consumption (of all size classes) and consumption of harvestable sized fish relative total (commercial+recreational) human consumption. The last two columns show the estimated value (k€) of the cormorants consumption of harvestable fish estimated from wholesaler list prices for commercial fisheries and average consumer surplus per kilogram fish in recreational fisheries.

Assuming cormorants feed on yellow eels and humans only catch silver eels.

The estimated decrease in human catches of these stocks by cormorants (ignoring potential compensatory effects like mortality and fishing effort) thereby also differed between fish species and archipelagos (Table 2). Despite cormorants mainly fed on flounder somewhat smaller than harvestable size in fisheries (Fig. 1), the human harvest of flounder was estimated to decrease 35–41% due to cormorants, likely being within the interval 6–60%. The cormorant consumption of perch in Karlskrona archipelago was estimated to decrease fisheries yields by 9%, or at least likely <20%. Our estimate of the impact by cormorants on yields for the other stocks was ≤4%, likely not exceeding 10% (Table 2). For the stocks not detected in the cormorant diet (perch, pike, whitefish in Mönsterås archipelago), the maximum estimated impact on fisheries catches were <3% (Table 2).

**Table 2:** Estimated instantaneous mortality due to cormorant predation and yearly decrease of human catches in percent due to cormorant consumption on harvestable sized fish, and estimated indirect loss of harvestable sized fish through cormorants’ consumption on smaller individuals.

Karlskrona	M*_c_*	Decrease	Interval	*H_EQ_*	*%H_EQ_*	*H_PY_*	*%H_PY_*
Perch	0.19	8.5%	1.6–23%	87226	106%	43078	34% (19%)
Pike	0.04	2.0%	0.4–6.3%	85813	33%	63361	19% (8%)
Cod	0.07	3.3%	0.6–10%	NA	NA	NA	NA
Flounder	0.68	35%	5.9–58%	NA	NA	NA	NA
Herring	0.002	0.1%	0.01–0.2%	NA	NA	NA	NA
Eel	NA	NA	NA	14920	168%	NA	NA
Whitefish	0.01	0.5%	0.1–1.5%	4060	13%	4069	12% (4.7%)
Mönsterås							
Perch	0	0%	0–1.6%	1432	25%	2146	27% (13%)
Pike	0	0%	0–1.0%	0	0%	0	0%
Cod	0.04	1.7%	0.3–5.6%	NA	NA	NA	NA
Flounder	0.79	41%	6.9–63%	NA	NA	NA	NA
Herring	0.002	0.1%	0.01–0.2%	NA	NA	NA	NA
Eel	NA	NA	NA	3194	7%	NA	NA
Whitefish	0%	0%	0–2.8%	0	0%	0	0%

‘Decrease’ is the estimated percentage decrease in human catches due to direct competition from cormorants. ‘Interval’ shows the 95% interval of the estimated decrease from the Monte Carlo simulations. *H_EQ_* (Harvestable sized equivalent) is the estimated biomass (kg) impact of cormorant consumption on harvestable sized fish including both direct consumption on harvestable sized fish and consumption on smaller fish not reaching harvestable size. %*H_EQ_* is *H_EQ_* relative human catches. *H_PY_* is the estimated increase in biomass (kg) of human yields if there was cormorant predation on neither harvestable sized fish nor smaller fish. %*H_PY_* is the proportional decrease in human yields due to cormorant predation on fish of all size classes, the value in brackets is the estimated value assuming a doubling of natural mortality and halved somatic growth in absence of cormorants.

NA = Non-applicable (see Material and Methods).

### Indirect Effects

When including the cormorants’ consumption on smaller fish (ignoring compensatory effects in fish body growth and mortality), the estimated removal of harvestable sized fish by cormorants (*H_EQ_*) at least doubled (Table 1, 2). The estimated impacts of cormorants on fisheries catches (%*H_PY_*) more than four-folded compared to the estimated direct competition for perch, pike and whitefish (Table 2). Also when including a doubled natural mortality and halved body growth to account for potential compensatory effects, estimated impacts was still twice or larger, compared to the estimate of direct competition only (Table 2). The estimated impact of cormorants on silver eel escapement differed between the two archipelagos; 14.9 tonnes at Karlskrona and 3.2 tonnes at Mönsterås (Table 2). This is close to the estimated direct removal of eel biomass (13 and 2.6 tonnes, respectively, Table 1), and corresponds to 168% and 7% of the human catches (in biomass) in respective area.

### Economic Estimates

The total value of the cormorants’ consumption of the harvestable fish, estimated from the marginal income for commercial fisheries to wholesalers, was €304,000 and € 41,364 in Karlskrona and Mönsterås archipelago, respectively (Table 1). This corresponds to 13% and 3.2% of total value of commercial and recreational catches in respective archipelago. In term of estimated reduced value of commercial and recreational catches the reduction was €63,000 in Karlskrona archipelago, of which >80% was from perch and pike that are dominated (90%) by recreational fisheries. The reduced value of catches in human fisheries in Mönsterås archipelago was €25,700, for which the value of flounder comprised >95%.

## Discussion

Based on gut contents we estimated that the cormorants’ consumption was 10 and 44% relative to the fish biomass removal in coastal human fisheries for the two studied areas. The estimated decrease in fisheries catches due to cormorants is uncertain, but on average estimated to be <10% for all species except flounder (>30%). When including the indirect effects of cormorant predation on fish below harvestable size the estimated impact on fisheries catches multi-folded for the species confined to an archipelago; for example up to a 34% for perch in Karlskrona archipelago. This neglects any compensatory effects from cormorant predation on natural mortality and somatic growth. But we conclude that natural mortalities must probably more than double and somatic growth reduced by half in absence of cormorant predation in order for indirect effects of cormorant predation on human catches to be negligible. And at least perch, eel and also probably pike have declined the last decades in many parts of the Baltic Sea [7] resulting in density dependent effects currently are probably low, and hence, also compensatory effects are probably low in some areas. In all, the estimated impact of cormorants was lower on the commercially most important stocks (cod, herring, eel) and higher for stocks important in recreational fisheries (perch and pike).

Our results diverge somewhat from earlier studies suggesting a differentiation in fish sizes targeted by cormorants and fisheries [17], [19], [20]. The majority of fish consumed by cormorants was smaller than harvestable size in human fisheries. But for most of the stocks considered here (excluding eel and perch in Mönsterås) cormorants included a fair part of individuals close to or large enough to be harvestable in fisheries.

A high cormorant consumption relative to fisheries does not automatically mean there is competition over fish resources. For most stocks the estimated direct impact of cormorant predation on fisheries’ yields was well below 10% when considering predation on harvestable sized fish. The exception was flounder for which an abolition of cormorants consumption are estimated to increase catches in fisheries >30%. There are, however, several sources of uncertainty in these calculations. The diet study is based on several hundred stomachs from each area. But the interval of the relative contribution of some fish species to total biomass can be 50–100% of the estimated value [26]. Although the number of breeding birds is rather well documented we had to use estimates from other studies on number of foraging days of non-breeding and overwintering birds. The latter is likely to be variable between years due to weather conditions. Another source of uncertainty, which we could not address due to lack of documentation, is loss of cormorant predation due to kleptoparasitism by gulls, skuas, and sea eagles. Personal observations by local fishermen indicate kleptoparasitism could be high occasionally, of especially larger fish, which could render an underestimation of the real cormorant effect in our calculations. The estimates of recreational fishing are based on questionnaires and a downscaling to a finer spatial scale. Estimates on natural mortality and fishing mortality come from published information or independently collected data but still remain uncertain or may not be representative for these areas.

To address these sources of uncertainty we did Monte Carlo simulations to get a range of values for the probable impact of cormorants on human catches. Albeit 95% intervals spanned over an order of magnitude, we can be fairly sure that in these archipelagos the direct competition is largest for flounder, likely being in the 6–60% interval. For perch in Karlskrona archipelago the predicted direct impact of cormorants on fisheries is likely in the order of 2–23%, while for the other fishes most likely <10%. Thus, flounder and perch seem to be the species investigated here for which there is a potential direct conflict between humans and cormorants for harvestable sized fish.

Cormorants also consume smaller sized fish that will not be recruited into harvestable sizes which may impact human catches of the more sedentary species, perch, pike and whitefish. The estimated increase in fisheries yields due to an abolition of cormorant predation increased at least four-fold when accounting for this indirect effect of cormorant fish predation (Table 2). However, these estimates ignore any compensatory mechanisms, e.g. reduced survival and body growth of an absence of cormorant predation, and therefore most likely overestimated. Somatic growth, of for example perch, in the Baltic Sea has been observed to be 25–35% lower in high density populations compared to low density populations [38]. Estimating changes in natural mortality with density in natural populations is difficult as densities are not independent of mortality rates. For example, a correlative study suggests a negative relation between mortality and density of perch [38]. If we anyway assume a doubled natural mortality and a halved somatic growth rate in absence of cormorant predation, the estimated increase in human catches still doubled compared to when not accounting for predation on small sized fish. Unless the compensatory mechanisms are much stronger than reported from natural variation, the indirect effects of cormorants from feeding on small fish are likely as large, or larger, than the estimated direct competition over harvestable sized fish.

For these sedentary species the indirect effect of cormorants’ consumption on smaller individuals may be the largest source of competition with fisheries. Van de Valk et al. [22] suggested a similar effect of double crested cormorants (*Phalacrocorax auritus*) on yields in recreational fisheries of yellow perch (*Perca flavescens*) in Oneida Lake. Otherwise this indirect effect of bird, or mammalian, predation on small fish on fisheries seems rarely being accounted for. Given the sometimes high consumption of cormorants of small fish [17], [39], [40] and the magnitude of indirect effects estimated here, we encourage future studies to consider this impact on fish populations.

We used data from gut analyses here as we had no *a priori* expectations over which species there might be a potential conflict between cormorants and humans. And studies from other areas are required to make more general conclusions about impact of cormorants on human catches. It is important to stress that this is only one model quantifying the conflict between humans and wildlife using consumption data only. There are other aspects of the conflicts neglected here, like long-term interactions or if cormorants forage at gears. In cases where one is more interested in the conflict over a specific fish stock it is possible to derive more precise estimates of mortalities. Data from tagging program [39], [41], especially in more closed system, could provide more precise information about mortalities and growth rates or other compensatory effects of a specific species. Despite the uncertainties in parameter values, we stress the use of competition models as a complement to correlative studies to infer conflicts between humans and wildlife over shorter temporal scales.

We have assumed a constant fishing effort and fishing mortality rates. For recreational fishing this may be a reasonable assumption as available time may constrain fishing effort and there are few regulations. Commercial fisheries can in contrast be regulated and effort may change with densities. The eel fishery is highly regulated, as only a fixed number of licences can harvest a fixed amount each of migrating silver eels. Thus, cormorants hardly affect landings in eel fisheries. Instead cormorants can affect the escapement rate of silver eels from the local population, which in turn may affect yield per effort. Also catches of cod and herring are regulated, but for these species competition from cormorants is estimated to affect human yields well below 10%. Thus, cormorants are estimated to have limited impact on the economically most valuable stocks for the commercial fisheries studied here. The largest impact of cormorants on fisheries is estimated for the less regulated fisheries perch, pike, and flounder, of which the former two are mainly targeted by recreational fisheries.

The results showed both differences and similarities between the two archipelago areas. Cormorant consumption and impact on fisheries catches of herring and flounder was relatively similar between the two areas. Differences between study areas were large especially for fish species with local populations, like perch and pike. In other areas of the Baltic Sea there may also potentially be competition for fish species not present in any larger quantities here, for example pikeperch [42]. Thus, the results cannot directly be applied to other areas or extrapolated to larger spatial scales, as the degree of competition seems contingent on local fish community composition.

There seems to be few or no estimates of the economic impacts of cormorant predation on human fisheries of wild fish stocks. Here the estimated value of the cormorant predation differed between €40,000–300,000 per year for the two 50 km coastal study areas. That corresponds to 3–13% of total values of human catches. The estimated monetary value of the reduction in human catches due to cormorant predation was on average estimated to be €25,000–63,000 in the studied areas. But given the uncertainties in Table 2 (2–3 times larger/smaller than average values), the range is likely in the order €10,000–150,000. This regards only the direct competition of harvestable sized fish, which means that the reduction including predation on undersized fish is likely higher. The estimated value is contingent on the value of single/few species. In Mönsterås flounder comprised almost all the value, whereas in Karlskrona perch and pike were the species comprising most of the value. This highlights a problem as perch and pike are mainly targeted by recreational fisheries for which estimates of marginal values of catches are absent. Moreover, for perch and pike there is a substantial catch-release fishery (15% and 50%, respectively, of all recreational catches [9]), which we have not considered here. With more precise data on economical values of catches for a specific stock and area, uncertainties can be reduced.

A mere high estimated impact of cormorants on fisheries catches cannot tell whether cormorant and fisheries affect long term dynamics of these stocks. Both fisheries and cormorants may affect long term dynamics but recruitment rates are also dependent on other factors [7], [43]. Temporal variation in abiotic factors (water temperature, salinity), habitat, fishing effort, or fish communities may alter both long term population dynamics and the competition between cormorants and humans over fish resources. Depletion of one stock without reduced yields or cormorant predation will increase the competition over that stock, or competition for other stocks. For example, in Karlskrona archipelago cod yields have declined due to a depleted stock and seal damages of the gear and catches, which may force local coastal fishermen to start targeting perch and pike. Also changes in fisheries management, like bans of specific fisheries (gears and species) or size regulations could quickly alter the competition between humans and cormorants. For example, eel fisheries are being closed in Europe by regulation, potentially causing an increased fishing pressure on other species.

We here provide a framework for quantitative assessment of the the effects of cormorants on fisheries. The assessment here is associated with uncertainties but provides a tool, which can be modified or used with more precise data, to estimate competition between predator populations and humans over shared resources based on local data. This approach would constitute a significant complement to previous analysis based on temporal and spatial comparisons of stock sizes and structures, landings and cormorant population size.

## Supporting Information

Figure S1 **Maps over the studied archipelagos location and size of cormorant colonies within.**
(TIF)

Table S1 **Number of cormorants examined for gut content per month in the two archipelagos.**
(DOCX)

Table S2 **Estimated daily intake of fish by cormorants at different periods over the year and number of cormorants in the two studied archipelagos.**
(DOCX)

Table S3 **Population parameter estimates used in the calculations.**
(DOCX)

Table S4 **Data used for calculating mortality and body growth of perch, pike and whitefish.**
(XLSX)

File S1 **R-script used for calculating estimates of direct impacts from cormorants on human catches and 95% confidence intervals from catch data and mortality rates.**
(R) Click here for additional data file.
