# Supplementary material for: Estimating Competition between Wildlife and Humans–A Case of Cormorants and Coastal Fisheries in the Baltic Sea
Source: PLoS One. 2013 Dec 30;8(12):e83763. doi: 10.1371/journal.pone.0083763 (PMC3875482; doi:10.1371/journal.pone.0083763)
Supplement: Table S1 — Number of cormorants examined for gut content per month in the two archipelagos. (DOCX) [file pone.0083763.s002.docx]

**Table S1**: Number of cormorants examined for gut content per month in the two archipelagos.

| Karlskrona | Year | Month | Number of cormorants |
| --- | --- | --- | --- |
|  | 2009 | August | 49 |
|  |  | September | 29 |
|  |  | October | 21 |
|  |  | November | 1 |
|  |  | December | 8 |
|  | 2010 | March | 40 |
|  |  | April | 7 |
|  |  | June | 3 |
|  |  | July | 36 |
|  |  | August | 61 |
|  |  | September | 28 |
|  |  | October | 12 |
| Mönsterås | 2009 | March | 19 |
|  |  | April | 54 |
|  |  | May | 37 |
|  |  | June | 29 |
|  |  | July | 34 |
|  |  | August | 30 |
|  |  | September | 14 |
|  |  | October | 12 |
