# Supplementary material for: Estimating Competition between Wildlife and Humans–A Case of Cormorants and Coastal Fisheries in the Baltic Sea
Source: PLoS One. 2013 Dec 30;8(12):e83763. doi: 10.1371/journal.pone.0083763 (PMC3875482; doi:10.1371/journal.pone.0083763)
Supplement: Table S2 — Estimated daily intake of fish by cormorants at different periods over the year and number of cormorants in the two studied archipelagos. (DOCX) [file pone.0083763.s003.docx]

**Table S2**: Estimated daily intake of fish by cormorants at different periods over the year and number of cormorants in the two studied archipelagos (Kk = Karlskrona, Mn = Mönsterås). The daily intake rates during the breeding period include fish for chicks and juveniles. The number of post-breeding cormorants includes both adult and juvenile birds.

| \| Period \| Incubation \| Small chicks \| Downy chicks \| Fledgings \| Post-breeding \| \| --- \| --- \| --- \| --- \| --- \| --- \| \| Daily intake (g) \| 238 \| 316 \| 588 \| 1080 \| 540 \| \| Date \| 1 Apr -  30 Apr \| 1 May –  10 May \| 11 May-  20 June \| 21 June-30 Sept \| 1 Oct-  31 March \| \| Days \| 30 \| 10 \| 40 \| 100 \| 185 \| \| Cormorants (Kk) \| 5250 \| 5250 \| 5250 \| 5250 \| 1090 \| \| Cormorants (Mn) \| 4500 \| 4500 \| 4500 \| 4500 \| 415 \| |  |  |  |  |  |  | |  |  |  |  | |  |  |  |  |
| --- | --- | --- | --- | --- | --- | --- | --- | --- | --- | --- | --- | --- | --- | --- | --- | --- | --- | --- | --- | --- | --- | --- | --- | --- | --- | --- | --- | --- | --- | --- | --- | --- | --- | --- | --- | --- | --- | --- | --- | --- | --- | --- | --- | --- | --- | --- | --- | --- | --- | --- | --- | --- |
|  |  |  |  |  |  |  |  |  |  |  |  |  |  |  |  |  |
