# Supplementary material for: Estimating Competition between Wildlife and Humans–A Case of Cormorants and Coastal Fisheries in the Baltic Sea
Source: PLoS One. 2013 Dec 30;8(12):e83763. doi: 10.1371/journal.pone.0083763 (PMC3875482; doi:10.1371/journal.pone.0083763)
Supplement: Table S3 — Population parameter estimates used in the calculations. (DOCX) [file pone.0083763.s004.docx]

**Table S3**: Population parameter estimates used in the calculations. *M_n_* is the estimated instantaneous natural mortality rate to other factors than cormorants and fishing, *F* instantaneous fishing mortality, *w_H_* and *T* average individual weight (kg) and age at minimum harvestable size. For eel *M_n_* is calculated per centimetre and *T* is length because of the large variation of age-specific length in silver eels. There are almost no yellow eels larger than 70 cm in these areas (Dekker et al. 2011).

| Karlskrona | *M_n_* | *F* | *w_H_* | *T* |
| --- | --- | --- | --- | --- |
| Perch^1^ | 0.5 | 0.2 | 0.1 | 3 |
| Pike^1^ | 0.5, 0.3* | 0.2 | 0.4 | 3 |
| Cod^2^ | 0.2 | 0.25 | 0.5 | NA |
| Flounder^3^ | 0.2 | 0.2 | 0.1 | NA |
| Herring^2^ | 0.2 | 0.25 | 0.04 | 2 |
| Eel^4^ | 0.05 | NA | 0.7 | 70 cm |
| Whitefish^1^ | 0.2 | 0.2 | 0.25 | 3 |
| Mönsterås | |  |  |  |
| Perch^1^ | 0.2 | 0.5 | 0.1 | 2 |
| Pike^1^ | 0.2 | 0.2 | 0.4 | 3 |
| Cod^2^ | 0.2 | 0.25 | 0.5 | NA |
| Flounder^3^ | 0.2 | 0.2 | 0.1 | NA |
| Herring^2^ | 0.2 | 0.25 | 0.04 | 2 |
| Eel^4^ | 0.05 | NA | 0.7 | 70 cm |
| Whitefish^1^ | 0.1 | 0.2 | 0.25 | 3 |

NA = Non-applicable.

*For pike we used a lower *M_n_* for sizes > *w_H_*.

1. Swedish Agency for Marine and Water Management. 2012b. Kustfiskedatabas. Retrieved 2 May from <https://fivbi.havochvatten.se/analytics/saw.dll?PortalPages>. Data available in Supplement Material S5.
2. ICES. 2011. Report of the Baltic fisheries assessment working group (WGBFAS). Retrieved 2 May from http://www.ices.dk/workinggroups/ViewWorkingGroup.aspx?ID=42.
3. ICES. 2010. Report of the ICES/HELCOM workshop on flatfish in the Baltic Sea (WKFLABA). Retrieved 2 May from <http://www.ices.dk/workinggroups/ViewWorkingGroup.aspx?ID=489>.
4. Dekker, W., H. Wickström, and J. Andersson. 2011. Status of the eel stock in Sweden in 2011. Aqua Reports 2011:2. Swedish University of Agricultural Sciences, Drottningholm, Sweden.
